# Factors Associated with In-Hospital Delay in Intravenous Thrombolysis for Acute Ischemic Stroke: Lessons from China

**DOI:** 10.1371/journal.pone.0143145

**Published:** 2015-11-17

**Authors:** Qiang Huang, Qing-feng Ma, Juan Feng, Wei-yang Cheng, Jian-ping Jia, Hai-qing Song, Hong Chang, Jian Wu

**Affiliations:** 1 Department of Neurology, Xuanwu Hospital, Capital Medical University, Beijing, China; 2 Department of Nursing, Xuanwu Hospital, Capital Medical University, Beijing, China; University of Pittsburgh, UNITED STATES

## Abstract

In-hospital delay reduces the benefit of intravenous thrombolysis (IVT) in acute ischemic stroke (AIS), while factors affecting in-hospital delay are less well known in Chinese. We are aiming at determining the specific factors associated with in-hospital delay through a hospital based cohort. In-hospital delay was defined as door-to-needle time (DTN) ≥60min (standard delay criteria) or ≥75% percentile of all DTNs (severe delay criteria). Demographic data, time intervals [onset-to-door time (OTD), DTN, door-to-examination time (DTE), door-to-imaging time (DTI), door-to-laboratory time (DTL) and final-test-to-needle time (FTN, the time interval between the time obtaining the result of the last screening test and the needle time)], medical history and additional variables were calculated using Mann-Whitney U or Pearson Chi-Square tests for group comparison, and multivariate linear regression analysis was performed to identify independent variables of in-hospital delay. A total of 202 IVT cases were enrolled. The median age was 61 years and 25.2% were female. The cutoff points for the upper quartile of DTN (severe delay criteria) was 135min.When compared with the reference group without in-hospital delay, older age, shorter OTD and less referral were found in the standard delay group and male sex, presence with transient ischemic attacks or rapidly improving symptom, and with multi-model CT imaging were more frequent in the severe delay group. In the multivariate linear regression analysis, FTN (*P*<0.001) and DTL (*P =* 0.002) were significantly associated with standard delay; while DTE (*P =* 0.005), DTI (*P =* 0.033), DTL (*P*<0.001), and FTN (*P*<0.001) were positively associated with severe delay. There was not a significant change in the trend of DTNs during the study period (*P* = 0.054). In-hospital delay was due to multifactors in China, in which time delays of decision-making process and laboratory tests contributed the most. Efforts aiming at reducing the delay should be focused on the optimization for the items of screening tests and improvement of the pathway organization.

## Introduction

Intravenous thrombolysis (IVT) with recombinant tissue plasminogen activator is one of the effective but time dependent therapies for acute ischemic stroke (AIS)[1,2]. However, in China, there were less than 22% of all AIS arriving at hospital within 3h, of which only 1.6% could be treated with IVT after series of screening tests [3]. In-hospital delay contributed substantially to the barriers of the availability to IVT, which could even jeopardize the population benefit from extending the therapeutic time window of IVT [4]. Programs addressing this problem have been launched in developed countries and achieved notable improvement in reducing door-to-needle time (DTN) of IVT and other major clinical endpoints such as mortality and hemorrhagic complications[5,6,7]. There are significant variations in Chinese healthcare system to those of western countries [8]. For example, tertiary hospitals and emergency medical service (EMS) in China are publicly owned but profit-driven, receive very limited financial support from the government and operate separately from each other. This might bring into a different profile for medical staff and policy maker focused on this area. We are aiming at determining the specific factors associated with in-hospital delay and sharing our experience in China through a hospital based cohort.

## Methods

### Ethics statement

The study protocol and data analysis were approved by the Ethical Committee of Xuanwu Hospital, and with the Declaration Helsinki. Written informed consents were obtained from all included patients or their proxies.

### Participants’ eligibility and enrollment

Consecutive AIS patients who were treated with IVT in a teaching hospital from March 2011 to December 2014 were included in this analysis. A neurological unit inside the emergency department (ED), an independent and trained stroke team (ST) and furnished stroke unit wards consisted of the major items of the pathway for IVT in AIS. For short, the nurse activated the pathway, and the neurologist at the ED started the screening until the ST member took the rest of the task. Time points of the flowchart for patient’s journey in our center were shown in S1 Fig. The pathway is available for 7 days a week and 24h a day. Conferences for monitoring the quality of the pathway were held monthly within departments of neurology and neurosurgery. Routine tests before IVT included head CT imaging, blood laboratory tests [blood cell counts, coagulation function and biochemistry tests (including serum electrolytes, renal and hepatic function)] and electrocardiogram. The inclusions and exclusions of IVT candidates followed the published Chinese guidelines[9], and extensive indications for IVT such as older than 80 years, onset-to-needle time (OTN) >4.5h but with penumbra in multi-model imaging [CT perfusion (CTP) or MR perfusion (MRP) imaging], mild stroke severity [as measured by the NIH stroke scale (NIHSS)], rapidly improving symptoms (RIS), baseline blood pressure ≥185/110mmHg, and so on might be also considered for IVT when other conditions were qualified. When the OTN of a candidate was predicted to be out of the 4.5h therapeutic window, a multi-model imaging (mainly CTP) could be employed and mismatch between mean transit time (MTT) and cerebral blood volume (CBV) was accepted as penumbra [10]. Buccal captopril and (or) intravenous urapidil was used as urgent management of rising blood pressure in order to bring gently the pressure below 185/110 mmHg. Altepalse (Boehringer-Ingelheim, Germany) was used as thrombolytic agent, and administrated strictly in accordance with recommendations [9].

### Explanatory and outcome variables

Demographic data (including sex, age, body mass index, medical insurance status, address, mode of transferring), baseline variables (NIHSS, blood pressure and sugar), medical history [hypertension, diabetes, dyslipidemia, coronary heart disease, atrial fibrillation and prior stroke], smoking and drinking status, drug history as well as additional factors likely to be associated with in-hospital delay [such as with urgent blood pressure management or multi-model imaging, present with transient ischemic attack (TIA) or RIS, lesion sites (classified as anterior and posterior circulation according to the results of later MR imaging), admission date and hour] were prospectively collected in forms of case reports by ST members. Variables of medical insurance status and medical history were dichotomized. Admission date was divided into working day and official holiday, while admission hour into working hours (from 8:00 to 18:00) and nonworking hours (from 18:00 to 8:00 at the second day).

### Related definitions

Stroke symptom onset was defined as the time when stroke symptoms first occurred or last time known to be normal. Door time was defined as when the patient arrived at the ED of the hospital, and needle time as when the administration of alteplase started. Referral was indicated if the patient got at least a heading CT imaging during his/her transferring, and the decision on whether to repeat the test(s) or not was made by ST member on duty. DTN and onset-to-door time (OTD) consisted of OTN. Time intervals between door time to the time of physical examination by ST, head imaging and last result from blood tests were defined as door-to-examination time (DTE), door-to-imaging time (DTI), and door-to-laboratory time (DTL), respectively. Final-test-to-needle time (FTN) was defined as the time interval between the last test (head imaging or laboratory test) and the needle time. FTN mainly contained the communicating process between ST and the patient or proxies for the IVT decision (decision-making process) and it could be 0 when the IVT decision was made before the last test finished. DTN was set as the primary endpoint of in-hospital delay as defined by DTN ≥60min (standard delay criteria) or ≥75% percentile of all DTNs (severe delay criteria).

### Statistical analysis

All calculations were performed using SPSS17.0 software, with two tailed *P* <0.05 as statistically significant. The continuous variables tested to be non-normal distribution with Kolmogorov–Smirnov test were presented as median and interquartile range (IQR). Mann-Whitney U test and Pearson Chi-Square test were used for related variables. For two types of in-hospital delay definitions (standard and severe delay criteria), with (defined as 1) and without (defined as 0) in-hospital delay were treated as dependent variables in the linear regression models, respectively. Multivariable linear regression analysis was used to identify factors of in-hospital delay in variables selected from the univariate analysis with a significance level ≤0.20. Exploratory analysis was conducted to detect the difference of DTNs during the 4-year period using Analysis of Variance (ANOVA) test.

## Results

### Patients’ characteristics

A total of 202 IVT cases were recruited, within whom one was treated with IVT twice for a recurrent AIS within 3 years. The median age was 61 (IQR 51–69) years, and 25.2% of the included subjects were female. The median NIHSS at enrollment was 9 (IQR 5–12). Less than 10%(20/202) had a DTN<60min, and 75% percentile of the DTN held a cutoff of 135min. Demographics and baseline characteristics are outlined in Table 1. Off-label IVTs were present in 46 cases (22.8%) with OTN >4.5h, 27(13.4%) with baseline blood pressure ≥185/110mmHg, 10 (5.0%) with mild stroke (defined as NIHSS <4), 8(4.0%) with RIS, 5 (2.5%) older than 80 years. Off-label IVTs became more frequent with time (S2 Fig).

**Table 1:** Baseline characteristics of included cases.

	Total (n = 202)	Standard delay criteria			Severe delay criteria		
		<60min(n = 20)	≥60min(n = 182)	*P*	<135min(n = 150)	≥135min(n = 52)	*P*
Age,y,	61(51–69)	52(46–65)	61(52–70)	0.046	61(51–67)	62(50–74)	0.484
Female	51(25.2)	4(20.0)	47(25.8)	0.569	31(59.6)	20(13.3)	0.011
Medical history							
Hypertension	122(60.4)	14(70.0)	108(59.3)	0.355	90(59.2)	32(61.5)	0.845
Diabetes	55(27.2)	6(30.0)	49(26.9)	0.792	40(26.3)	15(28.8)	0.761
Dyslipidemia	75(37.1)	5(25.0)	70(38.5)	0.237	57(37.5)	18(34.6)	0.663
CHD	30(14.9)	4(20.0)	26(14.3)	0.726	22(14.5)	8(15.4)	0.900
AF	32(15.8)	5(25.0)	27(14.8)	0.237	24(15.8)	8(15.4)	0.917
Prior stroke	39(19.3)	5(25.0)	34(18.7)	0.703	28(18.4)	11(21.2)	0.695
Current smoke	111(55.0)	10(50.0)	101(55.5)	0.639	85(55.9)	26(50.0)	0.405
Heavy drinking	69(34.2)	6(30.0)	14(7.7)	0.680	55(36.2)	14(26.9)	0.202
Baseline variables							
NIHSS	9(5–12)	10(5–13)	8(5–12)	0.391	9(5–12)	8(5–11)	0.632
SBP(mmHg)	150(130–165)	154(130–174)	149(130–165)	0.285	145(130–164)	150(131–170)	0.180
DBP(mmHg)	85(80–92)	90(80–98)	82(80–92)	0.249	85(80–95)	80(79–91)	0.334
Blood sugar (mmol/l)	6.9(5.8–8.7)	6.6(6.1–8.4)	7.1(5.8–8.8)	0.678	6.8(5.8–8.5)	7.1(5.9–9.7)	0.394
BMI	25.0(23.2–27.4)	26.9(23.7–29.7)	25.0(23.0–27.3)	0.080	25.0(23.1–27.4)	25.3(23.3–27.9)	0.802
Other variables							
Urgent management of BP	27(13.4)	3(15.0)	24(13.2)	1.000	19(12.5)	8(15.4)	0.620
Present as TIA or RIS	29(14.4)	2(10.0)	27(14.8)	0.803	15(9.9)	14(26.9)	0.003
Referral	70(34.7)	11(55.0)	59(32.4)	0.044	57(37.5)	13(25.0)	0.090
Lesion in AC	167(82.7)	19(95.0)	148(81.3)	0.221	125(82.2)	42(80.8)	0.674
Multi-model imaging	59(29.2)	5(25.0)	54(29.7)	0.663	36(23.7)	23(44.2)	0.003
Medical insurance	113(55.9)	11(55.0)	102(56.0)	0.929	87(57.2)	26(50.0)	0.317
Working days	147(72.8)	15(75.0)	132(72.5)	0.814	113(74.3)	34(65.4)	0.165
Working hours	114(56.4)	10(50.0)	104(57.1)	0.541	83(54.6)	31(59.6)	0.592

If not otherwise stated, continuous data are presented as median (IQR), *P* values were calculated using Mann-Whitney U test for continuous variables and Pearson Chi-Square test for categorical variables. IQR indicates interquartile range; in-hospital delay, in-hospital delay; NIHSS, National Institutes of Health Stroke Scale; CHD, coronary heart disease; AF, atrial fibrillation; SBP, systolic blood pressure; DBP, diastolic blood pressure; BP, blood pressure; BMI, body mass index; TIA, transient ischemic attack; RIS, rapidly improving symptoms; AC, anterior circulation.

When the standard delay criteria (DTN≥60min) was employed, the group with in-hospital delay was older (*P* = 0.046) and had lower rate in referral (*P* = 0.044) than the reference group (Table 1). When using the severe delay criteria (DTN≥135min), male sex (*P* = 0.011), present with TIA or RIS (*P* = 0.003), and multi-model imaging (*P* = 0.003) were more frequent in the in-hospital delay group (Table 1). All time intervals except for OTD were significantly longer in the in-hospital delay group (Table 2). As showed in table 2, in contrast to other time intervals, the OTDs were much longer in the non in-hospital delay group, though the comparison with severe delay didn’t reach a statistical significance (*P* = 0.083). Among the specific causes of time intervals, 87.6% (177/202) of DTLs were due to blood biochemistry tests (S1 Table).

**Table 2:** Time intervals of included cases.

Time intervals	Total(n = 202)	Standard delay criteria			Severe delay criteria		
		<60min(n = 20)	≥60min(n = 182)	*P*	<135min(n = 150)	≥135min(n = 52)	*P*
OTD(min)	110(67–164)	137(107–182)	100(65–160)	0.014	118(67–168)	90(62–130)	0.083
DTN(min)	116(93–135)	56(48–57)	120(101–138)	<0.001	105(86–120)	162(141–194)	<0.001
OTN(min)	229(185–270)	191(151–238)	232(187–274)	0.015	215(175–260)	260(228–323)	<0.001
DTE(min)	10(6–15)	7(5–10)	10(6–15)	0.017	10(5–13)	15(8–22)	0.003
DTI(min)	28(15–40)	12(0–21)	30(18–42)	<0.001	25(15–37)	36(22–61)	<0.001
DTL(min)	84(67–103)	59(40–90)	86(72–106)	0.006	82(67–96)	108(70–131)	<0.001
FTN(min)	27(11–45)	0(0–14)	30(15–46)	<0.001	22(8–37)	55(19–86)	<0.001

Data are presented as median (IQR), *P* values were calculated using Mann-Whitney U test for continuous variables and Pearson Chi-Square test for categorical variables. IQR indicates interquartile range; OTD, onset-to-door time; DTN, door-to-needle time; OTN, onset-to-needle time; DTE, door-to-examination time; DTI, door-to-imaging time; DTL, door-to-laboratory time; FTN, final-test-to-needle time.

### Univariate and multivariate analyses

As shown in Table 3, eight variables (OTD, DTI, DTL, FTN, age, body mass index, lesion in the anterior circulation and referral) and 11 variables (OTD, DTI, DTE, DTL, FTN, sex, referral, with CTP, present with TIA or RIS, admission date and medical insurance status) with *P* ≤0.20 in the univariate analysis (S2 Table) were included in the multivariate linear regression models, respectively. FTN (*P*<0.001) and DTL (*P =* 0.002) were significantly associated with standard in-hospital delay, while DTE (*P =* 0.005), DTI (*P =* 0.033) and aforementioned DTL and FTN (both with *P*<0.001) were positively associated with severe in-hospital delay. The exploratory ANOVA test indicated that there was no significant change in the trend of DTNs during the study period (*P =* 0.054) (S2 Fig).

**Table 3:** Multivariate Linear Regression Analysis to Identify Independent Variables that Affect In-hospital Delay.

Variables	Standard delay criteria		Severe delay criteria	
	Standardized coefficient	*P*	Standardized coefficient	*P*
Onset-to-door time	-.030	.684	.041	.515
Door-to-evaluation time	-	-	.171	.005
Door-to-imaging time	.127	.063	.142	.033
Door-to-laboratory time	.220	.002	.350	< .001
Final-test-to-needle time	.292	< .001	.548	< .001
Sex	-	-	.096	.088
Age	.096	.158	-	-
Body mass index	-.117	.084	-	-
Lesion in the AC	-.086	.193	-	-
Referral	-.066	.362	-.078	.193
CT perfusion imaging	-	-	.087	.143
Present as TIA or RIS	-	-	-.005	.939
Admission date	-	-	-.044	.415
Medical insurance status	-	-	-.020	.721

Standard delay criteria was defined as door-to-needle time ≥60min, while severe delay criteria defined as door-to-needle time ≥75% percentile of the DTNs. The null boxes indicated that the values were not enrolled in the multivariate analysis for *P* >0.200 in the univariate analysis; AC indicates anterior circulation; TIA, transient ischemic attack; RIS, rapidly improving symptoms.

## Discussion

We revealed the factors associated with in-hospital delay within the current healthcare system in China through a 4-year hospital based cohort. To our best knowledge, this is a facilitated work concerning in-hospital delay in Chinese population, which involves more time intervals and multiple factors. And the conservative definition (severe delay criteria) used in the present study accompany with the standard definition of in-hospital delay could help to identify the primary factors affecting DTN. In the primary analysis, time intervals including FTN and DTL consisted of the largest part of in-hospital delay, which implied that time delays of decision-making process for IVT and laboratory tests (mainly blood biochemistry tests) contributed the most of in-hospital delay. Other time intervals like DTE and DTI could also have contributed to severe in-hospital delay (DTN≥135min).

Time-sensitive decision-making process for thrombolytic therapy could be a factor of increased in-hospital delay, since the presentation of risk and benefit is often biased by the physicians who are under threat of frequent violence from the patients or their proxies indicated in a previous study [11] and not well understood by the general population [12]. Our study supported that the process of decision-making for IVT contributed to a prominent in-hospital delay, with a median FTN of 30min. The unique reasons for this phenomenon here may be as follows. Firstly, candidates of IVT in our country usually have more than one offspring, making it even harder to designate an appropriate decision-maker. Secondly, the hazard of increasing hemorrhagic complications was often exaggerated by physicians in clinical practice, especially for the elderly or MRIS cases [13], which might complicate the condition. Finally, expensive payment for IVT could also be one barrier of a fluent decision-making process. As for solutions, an informed outcome wheel [14] or other stroke tools [15], improvement in medical insurance and medical stuff training may facilitate the decision-making process and reduce DTN.

Time delays spent on laboratory tests were important elements of in-hospital delay and blood biochemistry tests played key roles. One previous study has indicated that waiting for the results of laboratory tests could cause unsubstantiated delay of IVT [16]. Our result also validated that DTL were independently associated with the in-hospital delay. According to our experience, excessive abnormal values of laboratory results were rare in patients without related previous history, which questioned the importance and necessity of various blood tests. Furthermore, DTI was also confirmed to be a factor of in-hospital delay in our study, consistent with other studies [17,18]. As for solutions to reduce the in-hospital delay of IVT, point-of-care (POC) laboratory test [19], all-points alarm or CT priority for head imaging [18,20], combination with two or many measures and optimal organization of these items (the Helsinki experience)[21] could work well. As we have known, the DTN in Helsinki University Central Hospital has been successfully reduced to be less than 20min after the improvement of the pathway organization [21]. Protocols aiming at addressing the delay of awaiting results of laboratory tests (especially when there are no specific clinical reasons) also could be preset to facilitate the decision-making process, which was recommended in the latest Canadian guideline[22].

There were some common negative indicators for in-hospital delay in our study. Old age and prior stroke were independent risk factors of in-hospital delay, consistent with a previous study [7]. The shorter OTD and the longer DTN were considered to be a deadline effect (or Parkinson law), which was also observed in other studies [7,17]. Present with TIA or RIS could have contributed to a complicated decision-making process for the unclear risk-benefit profile of IVT in mild stroke and RIS [23], which was also reported to increase DTN [7]. As for delay due to multi-model CT imaging, a learning curve of initial experience could be an explanation [24]. However, an Australian study showed in-hospital delay was associated with admission at non-working hour [25], which did not occur in our center probably due to a round-the-clock stroke team.

Notably, improvement programs mentioned above require substantial modifications to the present systems involving not only neurologists, but also many other departments (such as EMS, radiology, laboratory and so on). However, due to the significant differences of Chinese healthcare system[8], the situation of in-hospital delay for AIS showed in our study and the pre-hospital delay for AIS in urban China[26] were much worse than that in western countries[5,6]. It is noted that the independent factors associated with pre-hospital delay in urban China [26], which have little in common but could be internally correlated with those factors of in-hospital delay in our study, were visiting a local doctor before presenting at emergency, symptom onset at home, transfer to a Level III hospital for management, history of diabetes, hemorrhagic stroke, history of atrial fibrillation, unconsciousness at presentation, transfer by ambulance, and history of coronary artery disease. In fact, most hospitals in China don’t have POC tests for a single disease like stroke or an alert system for pre-notification in EMS at present. It is probably the primary reason why our median DTN did not change significantly in the 4-year study period, despite our continuous efforts made within our own departments. Moreover, even well designed randomized controlled trials with intensive implementation strategy could have faced a great difficulty in promoting IVT of AIS [27,28]. A recent progress made by the Stroke Emergency Mobile (STEMO) study [29] suggested that it was much better to put forward our frontline ahead and to integrate available resources.

### Limitations

Our study has limitations. Firstly, one single-center experience may not depict the full picture of the whole country. Secondly, we did not have a controlled group for comparison. Finally, our experience on reducing in-hospital delay might be only successfully applied in specific local conditions (e.g., Beijing). However, given to the huge homogeneity in the process of IVT, efforts to reduce in-hospital delay are more likely to work in a similar way.

## Conclusions

Our study demonstrated the factors affecting in-hospital delay in the present health system of China, which was due to multi-level factors and worth great efforts to reduce it. With shared experience of best practice, comprehensive quality improvement initiative (such as the optimization for the items of screening tests and improvement of the pathway organization) focused on specific factors affecting the in-hospital delay may serve as one of the best ways to reduce DTN.

## Supporting Information

S1 Fig Time points in patient’s journey in our center. ED indicates emergency department; IVT, intravenous thrombolysis.
(DOC)

S2 Fig Stem-and-leaf plots of median door-to needle time (DTN) in the 4-year study period. The analysis of variance test (ANOVA) showed a significance of 0.054 for comparison of DTNs.
(DOC)

S1 Table Items of the final laboratory tests for included cases (n = 202). (DOC)

S2 Table Univariate Linear Regression Analysis to Identify Independent Variables that Affect In-hospital Delay. Standard criteria of in-hospital delay was defined as door-to-needle time ≥60min, while severe delay criteria defined as door-to-needle time ≥75% percentile of the DTNs. NIHSS indicates National Institutes of Health Stroke Scale; AC, anterior circulation; TIA, transient ischemic attack; RIS, rapidly improving symptoms.
(DOC)
