# Supplementary material for: Factors Associated with In-Hospital Delay in Intravenous Thrombolysis for Acute Ischemic Stroke: Lessons from China
Source: PLoS One. 2015 Nov 17;10(11):e0143145. doi: 10.1371/journal.pone.0143145 (PMC4648585; doi:10.1371/journal.pone.0143145)
Supplement: S1 Fig — ED indicates emergency department; IVT, intravenous thrombolysis. (DOC) [file pone.0143145.s001.doc]

S1 Fig. Time points in patient’s journey in our center. (ED, emergency department; IVT, intravenous thrombolysis.)
