# Supplementary material for: Factors Associated with In-Hospital Delay in Intravenous Thrombolysis for Acute Ischemic Stroke: Lessons from China
Source: PLoS One. 2015 Nov 17;10(11):e0143145. doi: 10.1371/journal.pone.0143145 (PMC4648585; doi:10.1371/journal.pone.0143145)
Supplement: S2 Fig — The analysis of variance test (ANOVA) showed a significance of 0.054 for comparison of DTNs. (DOC) [file pone.0143145.s002.doc]

S2 Fig. Stem-and-leaf plots of median door-to needle time (DTN) in the 4-year study period (The analysis of variance test (ANOVA) showed a significance of 0.054 for comparison of DTNs)
