# Supplementary material for: Factors Associated with In-Hospital Delay in Intravenous Thrombolysis for Acute Ischemic Stroke: Lessons from China
Source: PLoS One. 2015 Nov 17;10(11):e0143145. doi: 10.1371/journal.pone.0143145 (PMC4648585; doi:10.1371/journal.pone.0143145)
Supplement: S1 Table — (DOC) [file pone.0143145.s003.doc]

S1 Table. Items of the final laboratory tests for included cases (n=202)

|  | Number of cases (%) |
| --- | --- |
| Blood cell counts | 4(2.0) |
| Coagulation function | 21(10.4) |
| Biochemistry tests | 177(87.6) |
