# Supplementary material for: Factors Associated with In-Hospital Delay in Intravenous Thrombolysis for Acute Ischemic Stroke: Lessons from China
Source: PLoS One. 2015 Nov 17;10(11):e0143145. doi: 10.1371/journal.pone.0143145 (PMC4648585; doi:10.1371/journal.pone.0143145)
Supplement: S2 Table — Standard criteria of in-hospital delay was defined as door-to-needle time ≥60min, while severe delay criteria defined as door-to-needle time ≥75% percentile of the DTNs. NIHSS indicates National Institutes of Health Stroke Scale; AC, anterior circulation; TIA, transient ischemic attack; RIS, rapidly improving symptoms. (DOC) [file pone.0143145.s004.doc]

| S2 Table. Univariate Linear Regression Analysis to Identify Independent Variables that Affect In-hospital Delay. (Standard criteria of in-hospital delay was defined as door-to-needle time ≥60min, while severe delay criteria defined as door-to-needle time ≥75% percentile of the DTNs. NIHSS indicates National Institutes of Health Stroke Scale; AC, anterior circulation; TIA, transient ischemic attack; RIS, rapidly improving symptoms.)   | **Variables** | **Standard criteria** | | **Conservative criteria** | | | --- | --- | --- | --- | --- | |  | **Standardized coefficient** | ***P*** | **Standardized coefficient** | ***P*** | | Onset-to-door time | -.149 | .034 | -.116 | .099 | | Door-to-evaluation time | .016 | .826 | .232 | .001 | | Door-to-imaging time | .200 | .004 | .323 | <.001 | | Door-to-laboratory time | .173 | .014 | .215 | .002 | | Final-test-to-needle time | .242 | .001 | .463 | <.001 | | Sex | -.040 | .572 | .179 | .011 | | Age | .142 | .044 | .050 | .480 | | NIHSS | -.026 | .717 | -.011 | .880 | | Body mass index | -.158 | .025 | .016 | .817 | | Blood sugar | .056 | .431 | .022 | .760 | | Systolic blood pressure | -.076 | .279 | .090 | .204 | | Diastolic blood pressure | -.060 | .394 | -.019 | .790 | | Urgent blood pressure control | -.016 | .822 | .035 | .622 | | Lesion in the AC | -.108 | .126 | -.030 | .616 | | CT perfusion imaging | .031 | .665 | .219 | .002 | | Recent TIA or MRIS | .041 | .561 | .211 | .003 | | Hypertension | -.065 | .357 | .037 | .602 | | Diabetes | -.021 | .771 | .021 | .762 | | Dyslipidemia | .083 | .239 | -.031 | .665 | | Coronary heart disease | -.048 | .498 | .009 | .901 | | Atrial fibrillation | -.083 | .239 | -.007 | .917 | | Prior stroke | -.048 | .499 | .028 | .697 | | Smoking | .033 | .641 | -.036 | .613 | | Heavy drink | .029 | .681 | -.066 | .351 | | Admission date | -.017 | .815 | -.098 | .167 | | Working hour | .043 | .543 | .061 | .392 | | Medical insurance status | .006 | .929 | -.093 | .187 | | Referral | -.142 | .044 | -.143 | .042 | |
| --- | --- | --- | --- | --- | --- | --- | --- | --- | --- | --- | --- | --- | --- | --- | --- | --- | --- | --- | --- | --- | --- | --- | --- | --- | --- | --- | --- | --- | --- | --- | --- | --- | --- | --- | --- | --- | --- | --- | --- | --- | --- | --- | --- | --- | --- | --- | --- | --- | --- | --- | --- | --- | --- | --- | --- | --- | --- | --- | --- | --- | --- | --- | --- | --- | --- | --- | --- | --- | --- | --- | --- | --- | --- | --- | --- | --- | --- | --- | --- | --- | --- | --- | --- | --- | --- | --- | --- | --- | --- | --- | --- | --- | --- | --- | --- | --- | --- | --- | --- | --- | --- | --- | --- | --- | --- | --- | --- | --- | --- | --- | --- | --- | --- | --- | --- | --- | --- | --- | --- | --- | --- | --- | --- | --- | --- | --- | --- | --- | --- | --- | --- | --- | --- | --- | --- | --- | --- | --- | --- | --- | --- | --- | --- | --- | --- | --- | --- | --- | --- | --- |
|  |
